# Derivation and validation of a model to predict acute kidney injury following cardiac surgery in patients with normal renal function

**DOI:** 10.1080/0886022X.2021.1960563

**Published:** 2021-08-09

**Authors:** Penghua Hu, Zhiming Mo, Yuanhan Chen, Yanhua Wu, Li Song, Li Zhang, Zhilian Li, Lei Fu, Huaban Liang, Yiming Tao, Shuangxin Liu, Zhiming Ye, Xinling Liang

**Affiliations:** a Division of Nephrology, The Affiliated Yixing Hospital of Jiangsu University, Yixing, China; b Division of Nephrology, Guangdong Provincial People’s Hospital, Guangdong Academy of Medical Sciences, Guangzhou, China; c The Second School of Clinical Medicine, Southern Medical University, Guangzhou, China

**Keywords:** Cardiac surgery, acute kidney injury, normal renal function, risk model

## Abstract

**Background:**

The study aimed to construct a clinical model based on preoperative data for predicting acute kidney injury (AKI) following cardiac surgery in patients with normal renal function.

**Methods:**

A total of 22,348 consecutive patients with normal renal function undergoing cardiac surgery were enrolled. Among them, 15,701 were randomly selected for the training group and the remaining for the validation group. To develop a model visualized as a nomogram for predicting AKI, logistic regression was performed with variables selected using least absolute shrinkage and selection operator regression. The discrimination, calibration, and clinical value of the model were evaluated.

**Results:**

The incidence of AKI was 25.2% in the training group. The new model consisted of nine preoperative variables, including age, male gender, left ventricular ejection fraction, hypertension, hemoglobin, uric acid, hypomagnesemia, and oral renin-angiotensin system inhibitor and non-steroidal anti-inflammatory drug within 1 week before surgery. The model had a good performance in the validation group. The discrimination was good with an area under the receiver operating characteristic curve of 0.740 (95% confidence interval, 0.726–0.753). The calibration plot indicated excellent agreement between the model prediction and actual observations. Decision curve analysis also showed that the model was clinically useful.

**Conclusions:**

The new model was constructed based on nine easily available preoperative clinical data characteristics for predicting AKI following cardiac surgery in patients with normal kidney function, which may help treatment decision-making, and rational utilization of medical resources.

## Introduction

Acute kidney injury (AKI) following cardiac surgery is a frequent complication with high mortality and medical costs [1]. Even a mild decline in renal function following cardiac surgery is independently allied with an increased risk of adverse cardiovascular events and chronic kidney disease [2,3]. Patients with renal insufficiency are more prone to AKI following cardiac surgery [4]. Hence, patients with renal insufficiency before surgery have attracted more attention in clinical practice. However, AKI following cardiac surgery is not an infrequent complication in patients with normal renal function with incidence ranging from 3.17% to 32.3% depending on different AKI diagnostic criteria and study populations [5–7]. Compared with the occurrence of AKI after cardiac surgery in patients with renal insufficiency, the occurrence of postoperative AKI in people with normal renal function is more devastating to patients and clinicians, which appears to be more unfavorable to the communication between doctors and patients. Tools for early diagnosis and risk assessment of postoperative AKI are needed to make clinical decisions and improve prognosis in these patients before surgery due to limited effective treatment options for AKI. With the increased use of electronic medical records, a clinical model based on routine data in electronic health records for AKI prediction has gradually gained more attention in recent years [8].

Several models for risk assessment of AKI following cardiac surgery have been identified and can be roughly classified into preoperative, intra-operative, or postoperative models [4,6,9–11]. Preoperative models are more feasible for clinical applications and can be utilized to identify high-risk patients who may benefit from preventive strategies such as hemodynamic management. The majority of preoperative models identified patients at risk of AKI requiring dialysis, which was a rare and late occurrence [4,10,11]. Some researchers have suggested that more effort is needed to develop a model for predicting any AKI stages [12]. Moreover, most previous models have focused on the risk of patients with renal insufficiency developing AKI after cardiac surgery [4,6,10,11]. The AKI risk factors appear to be different in patients with different baseline renal functions [13], which indicates that previous models may not be suitable for patients with normal renal function. However, the preoperative predictor data for this complication in individuals with normal renal function were limited. Hence, the present study sought to construct and validate a clinical model utilizing routinely collected preoperative data in electronic health records for risk assessment of AKI following cardiac surgery in individuals who had a normal renal function before surgery.

## Materials and methods

### Study population

The present study retrospectively evaluated the data for patients who underwent cardiac surgery using cardiopulmonary bypass at the Guangdong Provincial People’s Hospital (a tertiary teaching hospital) between 1 January 2006 and 31 December 2018 based on electronic medical records. Patients with normal renal function (estimated glomerular filtration rate [eGFR] of >60 ml/min × 1.72 m^2^ computed with the Chronic Kidney Disease-Epidemiology Collaboration formula [14]) were enrolled. The exclusion criteria were as follows: preoperative renal replacement therapy, history of unilateral nephrectomy, death during or within 24 h after the operation, serum creatinine value within 7 days after surgery was not available, cardiac transplantation, the critical state before surgery, or emergency surgery. Critical state before surgery is defined according to the European System for Cardiac Operative Risk Evaluation (Euroscore) II definition [15]. If patients underwent two or more procedures during the study period, only the first operation was analyzed. The study protocol was permitted by the Ethics Committee of Guangdong Provincial People’s Hospital without the need for signed informed consent from participants and complied with the ethical guidelines stated in the Declaration of Helsinki.

### Data collection and definition

Data on patient demographic characteristics, chronic comorbidities such as diabetes, hypertension, previous heart surgery, laboratory test results, medication administration records within a week before surgery, and procedure type were extracted from electronic health records. Baseline eGFR was computed with the Chronic Kidney Disease-Epidemiology Collaboration formula using the baseline serum creatinine level, which was established as the lowest creatinine level up to 3 months before hospital admission. If pre-admission creatinine value was unavailable, the minimum serum creatinine value during hospitalization before surgery was utilized. The magnesemia value less than 0.8 mmol/l was defined as hypomagnesemia.

### Outcome

Postoperative AKI was the outcome of the present study. The AKI definition was based on the Kidney Disease Improving Global Outcomes (KDIGO) criteria and included an elevation in serum creatinine of ≥0.3 mg/dl (26.5 μmol/l) within 2 days after surgery or serum creatinine increase of >1.5 times the baseline level within 7 days after surgery [16]. The urine output criteria were not used in the study since urine volume data were not available for most patients.

### Statistical analysis

Data were randomly split into training (70%) and validation (30%) groups. The continuous predictors (left ventricular ejection fraction (LVEF), hemoglobin, platelet, alanine aminotransferase, natremia, uric acid, albumin, low-density lipoprotein, and total bilirubin) were truncated at the first and 99th percentiles to limit the influence of extreme values [17]. Missing data were handled by multiple imputations using chain equations with an iteration of 20 times and merged according to Rubin’s rules [18]. Continuous variables were reported as median (interquartile range) or mean ± standard deviation and compared using Mann–Whitney *U* test or Student’s *t*-test. Categorical variables were presented as frequency (percentage) and compared using Fisher’s exact test. If Spearman’s correlation coefficient between variables was ≥0.40, only the variable judged to be more important on a clinical basis was included in the multivariate model [19]. The restricted cubic splines method was used to evaluate a possible nonlinear effect of the relationship between AKI risk and continuous variables [20]. Continuous variables were analyzed as categorical variables based on previously published literature or clinical expertise if needed [21].

We applied the least absolute shrinkage and selection operator (LASSO) regression to reduce the dimensions of the data and select variables in the training group. Ten-fold cross-validation and one-standard error rule were used to control for overfitting [22]. The final variables selected by LASSO were all included in the logistic regression analysis, and a new model was developed. To facilitate its clinical use, a nomogram was drawn based on the weight of each variable in the model.

The new model’s performance focusing on discrimination, calibration, and clinical usefulness was analyzed. The discrimination was evaluated using the area under the receiver operating characteristic curve (AUC). A calibration curve was drawn to evaluate the calibration and was accompanied by the Hosmer–Lemeshow test. The clinical value of the model was evaluated using decision curve analysis (DCA) calculated by quantifying the net benefits at different threshold probabilities in the validation group [23].

All analyses and reports for the development and validation of this model were compiled using the Transparent Reporting of a multivariable prediction model for Individual Prognosis Or Diagnosis guidelines. All analyses were computed using R software (version 3.6.1; https://www.r-project.org) and IBM SPSS v.25.0 (SPSS IBM, NY, USA). For analyses we used the R-packages (‘mice’, ‘VIM’, ‘corrplot’, ‘rms’, ‘glmnet’, ‘car’, ‘rms’, ‘ROCR’, and ‘rmda’). The R code for the analyses is also presented as Supplementary Appendix. Statistical significance was defined by the *p*-value of <0.05.

## Results

### Patient characteristics

**Figure 1.**
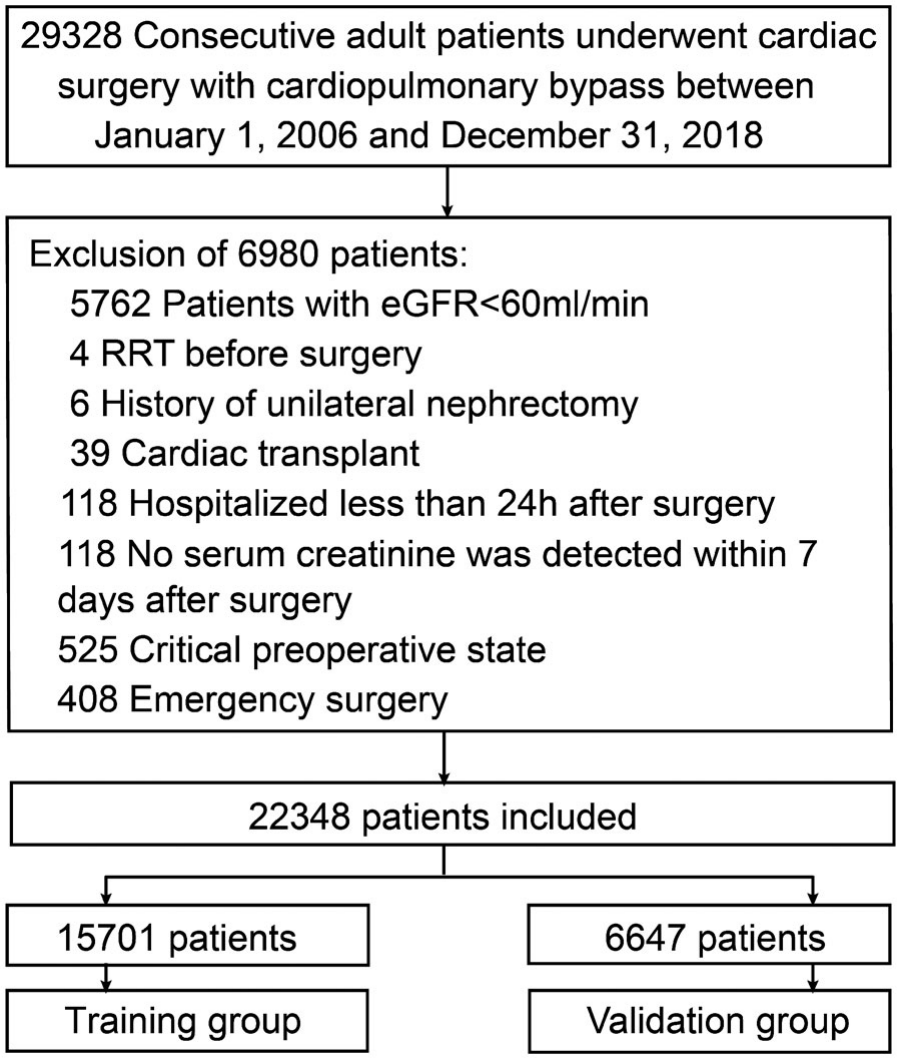
Participant selection flow chart. eGFR, estimated glomerular filtration rate; RRT, renal replacement therapy.

A total of 22,348 patients were included in the study, of which 15,701 patients were randomly selected for the training group and the remaining 6647 for the validation group. Figure 1 represents a detailed patient selection screening process. AKI incidence values in the training and validation groups were 25.2% (*n* = 3955) and 24.4% (*n* = 1621), respectively. The baseline characteristics of the study population are shown in Table 1.

**Table 1.** Baseline characteristics of training and validation groups.

Variables	Training group (*n* = 15,701)	Validation group (*n* = 6647)
Age, years	47.0 ± 14.2	47.0 ± 14.2
Male	7711 (49.1%)	3239 (48.7%)
LVEF, %	62.9 ± 9.4	62.8 ± 9.4
LVEF		
LVEF > 60%	11426 (72.8%)	4813 (72.4%)
40% < LVEF ≤ 60%	3851 (24.5%)	1649 (24.8%)
LVEF ≤ 40%	424 (2.7%)	185 (2.8%)
Baseline serum creatinine, µmol/l	75.5 ± 17.3	75.5 ± 17.2
eGFR, ml/min/1.73m^2^	94.2 ± 19.3	94.1 ± 19.0
Comorbidities		
Hypertension	3184 (20.3%)	1319 (19.8%)
Diabetes mellitus	791 (5.0%)	309 (4.6%)
Coronary heart disease	1504 (9.6%)	644 (9.7%)
COPD	227 (1.4%)	83 (1.2%)
Infectious endocarditis	848 (5.4%)	376 (5.7%)
Cerebrovascular disease	686 (4.4%)	305 (4.6%)
Peripheral vascular disease	65 (0.4%)	30 (0.5%)
Atrial fibrillation	4073 (25.9%)	1749 (26.3%)
PCI history	159 (1.0%)	69 (1.0%)
Previous cardiac surgery	454 (2.9%)	188 (2.8%)
History of transfusion	35 (0.2%)	16 (0.2%)
Recent contrast media exposure	3543 (22.6%)	1529 (23.0%)
Preoperative drugs use		
Renin-angiotensin system inhibitors	5261 (33.5%)	2219 (33.4%)
NSAID	2202 (14.0%)	903 (13.6%)
Aminoglycoside antibiotics	911 (5.8%)	364 (5.5%)
Stain	1945 (12.4%)	810 (12.2%)
Proton pump inhibitors	6458 (41.1%)	2745 (41.3%)
Erythrocyte transfusion, U	0.0 (0.0, 0.0)	0.0 (0.0, 0.0)
Procedure		
CABG	729 (4.6%)	312 (4.7%)
Valve	9790 (62.4%)	4166 (62.7%)
Aortic	1111 (7.1%)	486 (7.3%)
CHD	3391 (21.6%)	1393 (21.0%)
CABG + valve	456 (2.9%)	183 (2.8%)
Others	224 (1.4%)	107 (1.6%)
Laboratory Findings		
Hemoglobin, g/l	134.1 ± 19.1	134.2 ± 19.2
Platelet (×109/l)	205.1 ± 65.2	205.6 ± 65.1
Blood leucocytes (×109/l)	7.2 ± 2.6	7.2 ± 2.7
Natremia, mmol/l	139.0 ± 2.7	138.9 ± 2.7
Potassium, mmol/l	3.8 ± 0.4	3.8 ± 0.4
Magnesemia, mmol/l	0.9 ± 0.1	0.9 ± 0.1
Hypomagnesemia	3395 (21.6%)	1399 (21.0%)
Alanine aminotransferase, U/l	26.1 ± 28.9	27.8 ± 47.3
Uric acid, μmol/l	390.8 ± 113.4	390.2 ± 115.9
Albumin, g/l	37.9 ± 4.9	37.8 ± 4.9
International Normalized Ratio	1.2 ± 0.5	1.2 ± 0.4
Low density lipoprotein, mmol/l	3.0 ± 0.9	3.0 ± 1.0
Total bilirubin, μmol/l	19.1 ± 10.8	19.2 ± 10.8

CABG: coronary artery bypass grafting; CHD: congenital heart disease; COPD: chronic obstructive pulmonary disease; eGFR: estimated glomerular filtration rate; LVEF: left ventricular ejection fraction; NSAID: non-steroidal anti-inflammatory drugs; PCI: percutaneous coronary intervention.

### Predictors selection and model development

**Figure 2.**
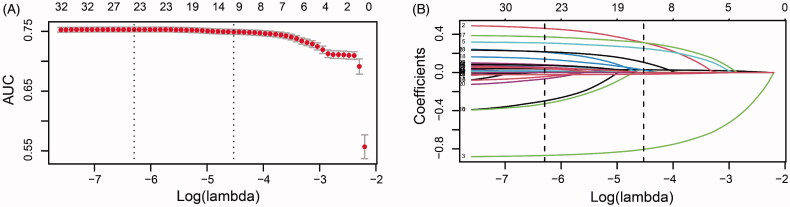
Predictor selection by the least absolute shrinkage and selection operator (LASSO) regression method. (A) The penalty tuning parameter (*λ*) in the LASSO model was conducted by ten-fold cross-validation with minimum criteria. Log(*λ*) was drawn vs. AUC. Dotted vertical lines were plotted at ideal values utilizing minimum criteria and one standard error of minimum criteria (1-SE criteria). Log(*λ*) of -4.528 and *λ* value of 0.0108 were selected. (B) Coefficient profile plot of the 35 predictors. Dotted vertical lines were plotted at ideal values utilizing the same criteria as in (A). Nine predictors with non-zero coefficients were selected.

**Figure 3.**
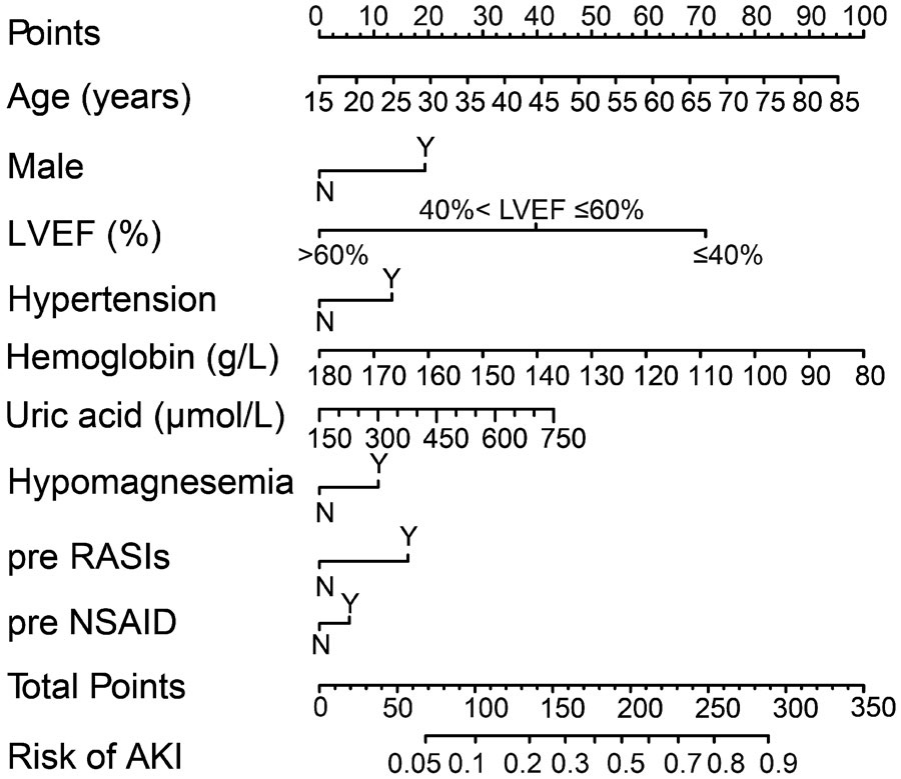
Nomogram for risk assessment of AKI after cardiac surgery in individuals who had normal kidney function. LVEF, left ventricular ejection fraction; NSAID, non-steroidal anti-inflammatory drugs; RASIs, renin-angiotensin system inhibitors.

Several variables were associated with increased risk of AKI according to the univariate analysis in the training group (online Supplementary Table S1). To generate the model for assessing the risk of AKI, a total of 35 potential predictors were contained in the LASSO regression, allowing to select nine best predictors: age, male gender, LVEF, hypertension, hemoglobin, uric acid, hypomagnesemia, and oral non-steroidal anti-inflammatory drug (NSAID) and renin-angiotensin system inhibitor (Figure 2). These variables were all included to develop a model using logistic regression. Table 2 presents detailed information on the variables in the final model. A nomogram was also drawn according to the logistic regression results (Figure 3).

**Table 2.** Multivariate logistic regression analysis of variables for predicting acute kidney injury after cardiac surgery.

variables	*β*	SE	*p*	OR	95%CI	
Age, years	0.032	0.002	<0.001	1.032	1.029	1.036
Male	0.452	0.044	<0.001	1.572	1.443	1.712
LVEF			<0.001			
LVEF > 60%				1		
40%< LVEF ≤ 60%	0.928	0.043	<0.001	2.529	2.324	2.753
LVEF ≤ 40%	1.656	0.109	<0.001	5.236	4.227	6.486
Hypertension	0.311	0.05	<0.001	1.364	1.238	1.504
Preoperative drugs use						
Renin-angiotensin system inhibitors	0.379	0.044	<0.001	1.461	1.341	1.593
NSAID	0.128	0.056	0.021	1.137	1.019	1.267
Hemoglobin, g/l	−0.023	0.001	<0.001	0.977	0.975	0.979
Hypomagnesemia	0.252	0.048	<0.001	1.286	1.172	1.412
Uric acid, μmol/l	0.002	0.000	<0.001	1.002	1.001	1.002
Constant	−1.065	0.179	<0.001			

LVEF: left ventricular ejection fraction; NSAID: non-steroidal anti-inflammatory drugs.

### Performance of the model

**Figure 4.**
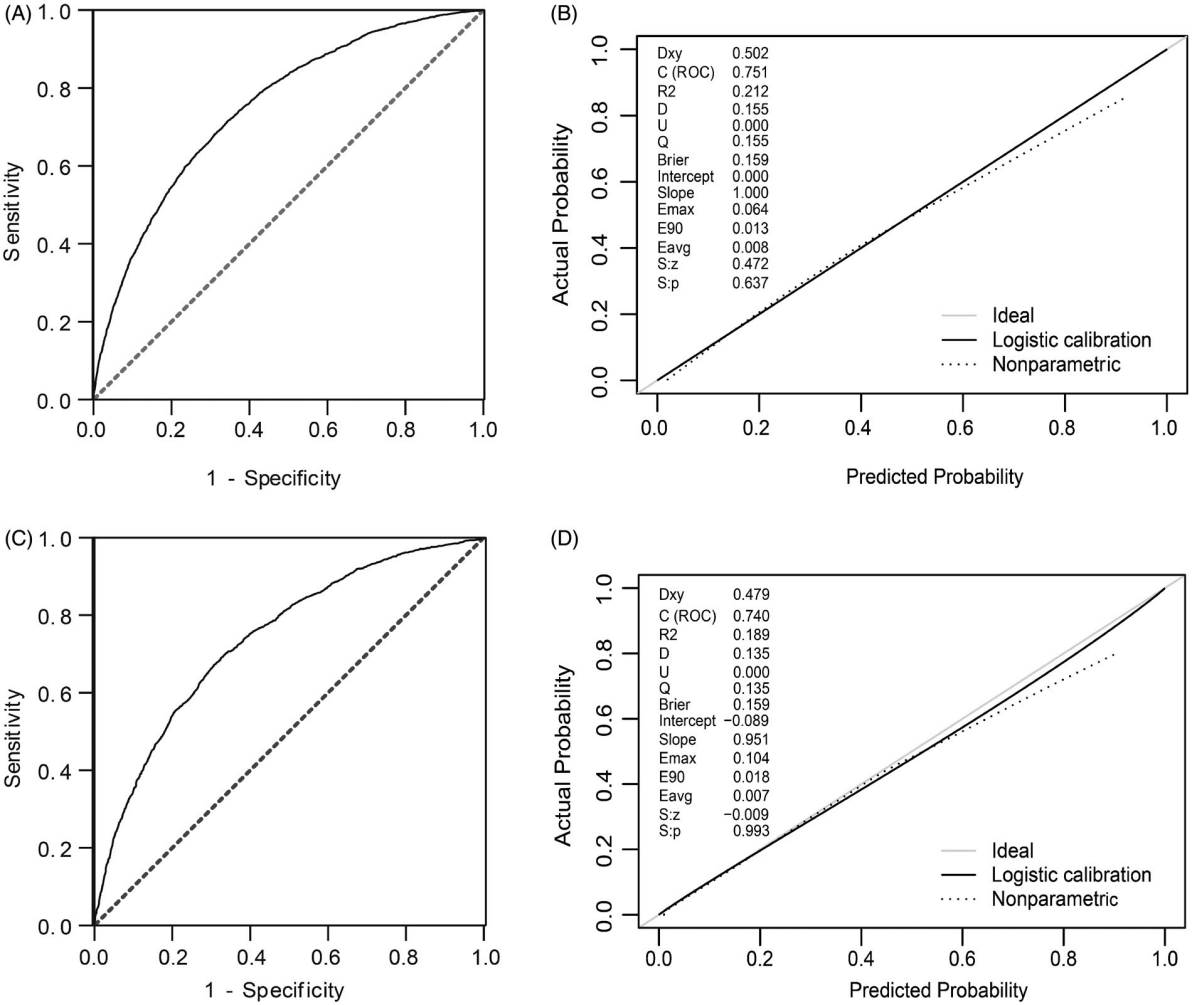
Model receiver operating characteristic and calibration curves. (A) AUC for postoperative AKI was 0.751 (95% CI, 0.743–0.760) in training group. (B) Calibration curve for new model in training group. (C) AUC for postoperative AKI was 0.740 (95% CI, 0.726–0.753) in validation group. (D) Calibration curve for new model in validation group. Calibration plots illustrate the relationship between the predicted AKI risk according to the models and actual occurrence of AKI in the validation data. Plot along the 45° line represents model calibration in which predicted probabilities are identical to actual outcomes. Dotted line has a close fit to solid line, indicating a better predictive model.

**Figure 5.**
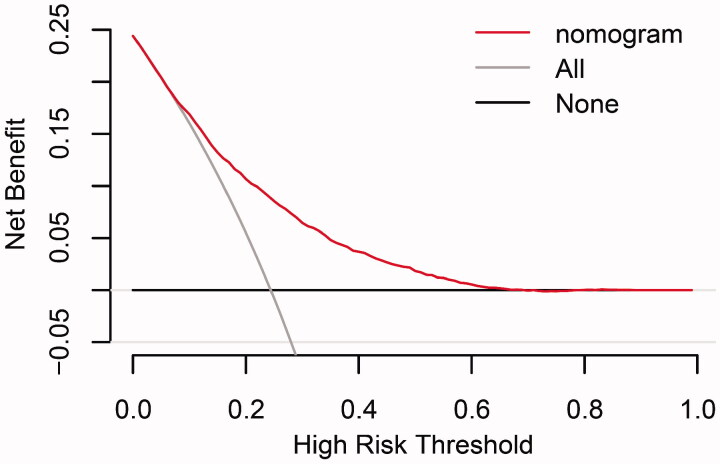
Decision curve analyses for prediction model. *X*- and *Y*‐axes show threshold probability and net benefit, respectively. Dashed and solid black lines represent the hypothesis that no patients and all patients had AKI, respectively. Net benefit was computed by subtracting the proportion of false positives from the proportion of true positives in all patients, weighting relative harm driven by the false positive. Threshold probability was estimated as the expected benefit of avoiding treatment equivalent to the expected treatment benefit. Net new model benefits are represented for each decision threshold. Using the new model to predict the risk of postoperative AKI generated a net benefit across most of the range of prediction thresholds.

The discrimination and calibration of the new model are presented in Figure 4. The model demonstrated good discrimination with an AUC of 0.751 (95% confidence interval (CI), 0.743–0.760) in the training group and 0.740 (95% CI, 0.726–0.753) in the validation group. The calibration plots represented an excellent agreement between actual observations and model prediction in the training and validation groups. The DCA curve indicated that using the new model for risk assessment of postoperative AKI generated a net benefit within most of the range of prediction thresholds (Figure 5).

## Discussion

The present study constructed and validated a model using easily available preoperative clinical data to predict the risk of AKI following cardiac surgery in individuals who had normal kidney function. The model included nine objectively measured variables: age, male gender, LVEF, hypertension, hemoglobin, and uric acid levels, hypomagnesemia, and oral renin-angiotensin system inhibitor and NSAID within 1 week before surgery. The model demonstrated good performance and was valuable across a range of threshold probabilities. A nomogram was also created to offer doctors a quantitative tool for predicting the individual risk of postoperative AKI.

The occurrence of AKI after cardiac surgery was associated with preoperative, intraoperative, and postoperative factors. The performance of the model developed using preoperative variables will be improved by adding the intraoperative or postoperative factors. However, the model developed using perioperative variables could not be used before surgery. The risk assessment of patients before surgery recommended by the National Institute for Health and Care Excellence (NICE) guidelines was more in line with the requirement of good clinical practice and easy to communicate with patients[24]. Fortunately, the predictive AKI model can be developed only using preoperative data [25]. Recently, some models for predicting AKI requiring dialyses, such as the Cleveland clinic score [11] and Mehta score [10], were developed using only preoperative data. However, the weight of renal insufficiency was high in these models, which limited use in people with normal renal function. Thus, the present study sought to construct a model utilizing routinely collected preoperative data for risk assessment of AKI after cardiac surgery in individuals who had a normal renal function before surgery. The new model demonstrated good discrimination with an AUC of 0.740 (95% CI, 0.726–0.753) in the validation group. The reason that the value of AUC was not high enough may be due to the model was developed only using the preoperative variables. Generally, the value of AUC above 0.7 is considered to be good predictive power [26].

Similar to previous findings [4,6,10,27,28], some conventional factors, such as age, male gender, LVEF, hypertension, and oral renin-angiotensin system inhibitor and NSAID before surgery were predictors of AKI. Furthermore, some factors, such as high uric acid, low hemoglobin, hypomagnesemia were also predictors in the present study.

High uric acid levels before surgery increased the risk of AKI in patients with cardiac surgery, which was consistent with previous findings [29]. Uric acid causes kidney damage through multiple mechanisms, including promoting the apoptosis of proximal renal tubules and vascular endothelial cells, activating the renin-angiotensin system to induce vasoconstriction, increasing reactive oxygen radical levels, and promoting the release of inflammatory mediators[30].

Low hemoglobin level was another AKI risk predictor. A study enrolling 6130 cardiac surgery patients showed the risk of AKI in patients with lower hemoglobin was increased by 29% [31]. The mechanism for that remains unclear. The underlying reasons may be related to several aspects. On one hand, low hemoglobin concentrations may cause renal hypoxia, resulting in oxidative stress increase, which renders the kidney susceptible to hypoxia and injury [32,33]. On the other hand, it may significantly increase the risk of intraoperative or postoperative RBC transfusion, which is an established risk factor for AKI [34].

Hypomagnesemia was also a significant contributing predictor for AKI. Magnesium may protect renal tissue from ischemia-reperfusion injury by protecting cell membrane lipid peroxidation, stimulating nitric oxide release, reducing arteriole tension, and increasing renal blood flow [35,36]. Furthermore, magnesium plays a significant role in regulating energy metabolism. Hypomagnesemia may lead to abnormal energy metabolism and exacerbate hypoxia, inducing AKI [37]. Hypomagnesemia represents an independent risk factor for AKI in hospitalized or tumor patients [38,39]. However, data on the correlation between hypomagnesemia and AKI in patients with cardiac surgery are limited and further research is still needed.

Baseline renal function has been confirmed to be an independent risk factor for AKI [4,10]. In the univariate analysis of the present study, baseline renal function in AKI patients was lower than that in non-AKI patients. However, unlike the previous studies [4,10], renal function was not selected in the final model. The possible reason may be that patients with normal renal function were included. The baseline renal function in the present study was better than that in previous studies.

AKI increased hospital mortality, prolonged hospitalization, and increased healthcare costs. Patients with normal renal function before surgery underwent AKI after cardiac surgery, which was devastating to both surgeon and patients. Though more frequent monitoring of kidney function in patients with surgery may facilitate early detection of kidney injury, doing so for all patients may waste medical resources. The model, visualized with the nomogram, for preoperative prediction of AKI may be beneficial for personalized therapies and clinical decision-making. The surgeon may inform the patients and family members about the risk of postoperative AKI and optimize treatment options before surgery, such as surgical method, frequency of renal function monitoring, and selection of antimicrobial drugs. For high-risk patients, correcting anemia, lowering uric acid, improving hypomagnesemia before surgery may reduce the occurrence of AKI and improve prognosis. Moreover, early intervention by nephrologists may be required for high-risk patients to strengthen renal function follow-up after discharge, so as to early detect the existence of chronic kidney disease. Finally, it may favor clinical studies to recruit suitable subjects and then evaluate the effect of interventions on renal function.

Some limitations also should be noted in the present study. First, despite the relatively large sample size, the data were limited to a retrospective single-center study with a relatively long duration. Evolution in treatment protocols, advances in anesthesia and surgical techniques, and changes in management strategies may have an impact on the occurrence of AKI. However, due to the lack of relevant data in this retrospective study, the model must be validated at other centers before it is widely used. Second, patients with low hemoglobin levels before surgery may have some underlying non-renal diseases, such as malignant tumors, gastric ulcers, and abnormal iron metabolism, which may cause kidney damage. Since the study was retrospective, these data were not available. BMI, preoperative beta-blockers, and preoperative vancomycin were also not available due to the same reason. The potential effect of these variables on our model was unclear. Finally, since urine output information was unavailable, only serum creatinine levels recommended by KDIGO were used as the diagnostic criterion for AKI, which may underestimate the rate of AKI.

In conclusion, a model was developed based on nine easily available preoperative clinical data characteristics for predicting the risk of AKI following cardiac surgery in patients with normal baseline renal function. The model may be beneficial for doctor-patient communication, treatment decision-making, and rational utilization of medical resources. In the future, the model will be integrated into the electronic medical records system to facilitate its clinical application.

## Supplementary Material

Supplemental Material

Supplemental Material

## Data Availability

The datasets used and analyzed during the current study are available from the corresponding authors on reasonable request.

## Abbreviations

AKI: acute kidney injury
eGFR: estimated glomerular filtration rate
Euroscore II: European System for Cardiac Operative Risk Evaluation II
KDIGO: Kidney Disease: Improving Global Outcomes
LVEF: left ventricular ejection fraction
LASSO: least absolute shrinkage and selection operator
AUC: area under the receiver operating characteristic curve
DCA: decision curve analysis
NSAID: nonsteroidal anti-inflammatory drugs
CABG: coronary artery bypass grafting
CHD: congenital heart disease
COPD: chronic obstructive pulmonary disease
PCI: percutaneous coronary intervention
NICE: National Institute for Health and Care Excellence
