# Supplementary material for: Derivation and validation of a model to predict acute kidney injury following cardiac surgery in patients with normal renal function
Source: Ren Fail. 2021 Aug 9;43(1):1205–13. doi: 10.1080/0886022X.2021.1960563 (PMC8354173; doi:10.1080/0886022X.2021.1960563)
Supplement: Supplemental Material [file IRNF_A_1960563_SM7613.pdf]

Supplemental materials: The R code for the analyses

```
##------- Data loading -------##
AKI_data <- read.csv("AKI_data.csv")


##---- Making traing and testing data sets      ----## set.seed(123)
#random number generator ind <- sample(2, nrow(AKI_data), replace
= TRUE, prob = c(0.7, 0.3)) train<- AKI_data[ind==1, ]          #the
training data set test <- AKI_data[ind==2, ]  #the testing data set
write.csv(train, file ="train.csv") write.csv(test, file ="test.csv")


##--Missing data and multiple interpolation in training set---##
install.packages("mice") install.packages("VIM")
library(mice) library("VIM") train1 <- read.csv("train.csv")
train1_preimp <- train1 for (i in
names(train1_preimp)[c(2,5:22,37)]) {
   train1_preimp[,i] <- as.factor(train1_preimp[,i])}
md.pattern(train1_preimp) aggr(train1_preimp, prop=T,numbers=T)
matrixplot(train1_preimp) set.seed(123) train1_preimp_imp <- mice(train1_preimp,
m=20) train1_preimp_imp impute <- function(x,
x.impute){ifelse(is.na(x),x.impute,x)} train1.imp <- train1 train1.imp$EF <-
impute(train1.imp$EF,
sum(train1_preimp_imp$imp$EF)/(sum(is.na(train1_preimp$EF))*20))
train1.imp$Hb <- impute(train1.imp$Hb,
                    sum(train1_preimp_imp$imp$Hb)/(sum(is.na(train1_preimp$Hb))*20))
train1.imp$PLT <- impute(train1.imp$PLT,
                    sum(train1_preimp_imp$imp$PLT)/(sum(is.na(train1_preimp$PLT))*20))
train1.imp$WBC <- impute(train1.imp$WBC,
                    sum(train1_preimp_imp$imp$WBC)/(sum(is.na(train1_preimp$WBC))*20))
train1.imp$NA. <- impute(train1.imp$NA.,
                    sum(train1_preimp_imp$imp$NA.)/(sum(is.na(train1_preimp$NA.))*20))
train1.imp$k <- impute(train1.imp$k,
                    sum(train1_preimp_imp$imp$k)/(sum(is.na(train1_preimp$k))*20))
train1.imp$MG <- impute(train1.imp$MG,
                    sum(train1_preimp_imp$imp$MG)/(sum(is.na(train1_preimp$MG))*20))
train1.imp$ALT <- impute(train1.imp$ALT,
                    sum(train1_preimp_imp$imp$ALT)/(sum(is.na(train1_preimp$ALT))*20))
train1.imp$UA <- impute(train1.imp$UA,
                    sum(train1_preimp_imp$imp$UA)/(sum(is.na(train1_preimp$UA))*20))
```

```
train1.imp$ALB <- impute(train1.imp$ALB,
                    sum(train1_preimp_imp$imp$ALB)/(sum(is.na(train1_preimp$ALB))*20))
train1.imp$INR <- impute(train1.imp$INR,
                    sum(train1_preimp_imp$imp$INR)/(sum(is.na(train1_preimp$INR))*20))
train1.imp$LDL <- impute(train1.imp$LDL,
                    sum(train1_preimp_imp$imp$LDL)/(sum(is.na(train1_preimp$LDL))*20))
train1.imp$TIDL <- impute(train1.imp$TIDL,
                    sum(train1_preimp_imp$imp$TIDL)/(sum(is.na(train1_preimp$TIDL))*20))

##---correlation between variables in training data---##
install.packages("corrplot") library(corrplot) data <- train1.imp data1 <- data[,-
37] corr <- cor(data1,method = "spearman") corrplot(cor(data1)) corrplot(corr =
corr,add=TRUE, type="lower", method="number",order="AOE",
col="black",diag=FALSE,tl.pos="n", cl.pos="n")

##--Restricted cubic splines---## install.packages("rms")
library(rms) ddist <- datadist(data) options(datadist='ddist')
fit.age <- lrm(AKI ~ rcs(age,3),data=data,x=TRUE,y=TRUE)
ggplot(Predict(fit.age, age, ref.zero=F)) fit.EF <- lrm(AKI
~ rcs(EF,3),data=data,x=TRUE,y=TRUE)
ggplot(Predict(fit.EF, EF, ref.zero=T))
fit.GFR.CKDEPI <- lrm(AKI ~
rcs(GFR.CKDEPI,3),data=data,x=TRUE,y=TRUE) ggplot(Predict(fit.GFR.CKDEPI,
GFR.CKDEPI, ref.zero=F)) fit.Hb <- lrm(AKI ~
rcs(Hb,3),data=data,x=TRUE,y=TRUE)
ggplot(Predict(fit.Hb, Hb, ref.zero=F))
fit.PLT <- lrm(AKI ~ rcs(PLT,3),data=data,x=TRUE,y=TRUE)
ggplot(Predict(fit.PLT, PLT, ref.zero=T))
fit.WBC <- lrm(AKI ~ rcs(WBC,3),data=data,x=TRUE,y=TRUE)
ggplot(Predict(fit.WBC, WBC, ref.zero=F)) fit.NA. <- lrm(AKI ~
rcs(NA.,3),data=data,x=TRUE,y=TRUE) ggplot(Predict(fit.NA.,
NA., ref.zero=T)) fit.k <- lrm(AKI ~
rcs(k,3),data=data,x=TRUE,y=TRUE) ggplot(Predict(fit.k, k,
ref.zero=T)) fit.MG <- lrm(AKI ~
rcs(MG,3),data=data,x=TRUE,y=TRUE) ggplot(Predict(fit.MG, MG,
ref.zero=T)) fit.ALT <- lrm(AKI ~
rcs(ALT,3),data=data,x=TRUE,y=TRUE) ggplot(Predict(fit.ALT,
ALT, ref.zero=T)) fit.UA <- lrm(AKI ~
rcs(UA,3),data=data,x=TRUE,y=TRUE) ggplot(Predict(fit.UA, UA,
ref.zero=F)) fit.ALB <- lrm(AKI ~
```

```
rcs(ALB,3),data=data,x=TRUE,y=TRUE) ggplot(Predict(fit.ALB,
ALB, ref.zero=F)) fit.INR <- lrm(AKI ~
rcs(INR,3),data=data,x=TRUE,y=TRUE) ggplot(Predict(fit.INR,
INR, ref.zero=F)) fit.LDL <- lrm(AKI ~
rcs(LDL,3),data=data,x=TRUE,y=TRUE) ggplot(Predict(fit.LDL,
LDL, ref.zero=F)) fit.TIDL <- lrm(AKI ~
rcs(TIDL,3),data=data,x=TRUE,y=TRUE) ggplot(Predict(fit.TIDL,
TIDL, ref.zero=T))

write.csv(data, file ="data.csv") data2 <- read.csv("data.csv")   ##---data: continuous variables
analyzed as categorical variables---##

##---Least absolute shrinkage and selection operator regression---##
install.packages("glmnet") library(glmnet) x=data.matrix(data2[,c(1:35)]) y
<- data2[,36] alpha1_fit <- glmnet(x,y,alpha=1,family="binomial")
plot(alpha1_fit,xvar="lambda",label=TRUE) set.seed(123) alpha1.fit <-
cv.glmnet(x,y,type.measure = "auc",alpha=1,family="binomial")
plot(alpha1.fit) print(alpha1.fit) lasso.coef <-
predict(alpha1.fit,type='coefficient',s=alpha1.fit$lambda.1se) lasso.coef
alpha1_fit <- glmnet(x,y,alpha=1,family="binomial")
plot(alpha1_fit,xvar="lambda",label=TRUE)
abline(v=log(c(alpha1.fit$lambda.min,alpha1.fit$lambda.1se)),lty=2)

##----logistic regression analysis with variables selected by LASSO---##
install.packages("car") library(car)
fit1 <- glm(AKI~age+male+EF+Hypertension.histroy+preACEI+Hb+UA+MG+preNSAID,
                    family = binomial, data = data2)
summary(fit1)

##--- nomogram---##
library(rms) ddist <-
datadist(data2)
options(datadist='ddist')
f1  <-  lrm(AKI~age+male+EF+Hypertension.histroy+preACEI+Hb+UA+MG+preNSAID,
data=data2,x=TRUE, y=TRUE) nom1 <- nomogram(f1, fun=plogis,fun.at=c(.001, .01, .05,
seq(.1,.9, by=.1), .95, .99, .999), lp=F, funlabel="Risk of AKI")
plot(nom1)
```

```
##---ROC in training data---## install.packages("ROCR")
library(ROCR) probs <- predict(fit1, newdata = data2,
type = "response") pred.full <- prediction(probs,
data2$AKI) perf.full <- performance(pred.full, "tpr",
"fpr") plot(perf.full, main = "ROC", col = "red")
performance(pred.full, "auc")@y.values

##---calibration curve in training data---##
val.prob(probs,data2$AKI)

##--Missing data and multiple interpolation in testing set---##
library(mice) library("VIM") test1 <- read.csv("test.csv")
test1_preimp <- test1 for (i in
names(test1_preimp)[c(2,5:22,37)]) {
   test1_preimp[,i] <- as.factor(test1_preimp[,i])}
md.pattern(test1_preimp)
aggr(test1_preimp, prop=T,numbers=T) matrixplot(test1_preimp) set.seed(123)
test1_preimp_imp <- mice(test1_preimp, m=20) test1_preimp_imp test1.imp <-
test1 impute <- function(x, x.impute){ifelse(is.na(x),x.impute,x)} test1.imp$EF <-
impute(test1.imp$EF,
sum(test1_preimp_imp$imp$EF)/(sum(is.na(test1_preimp$EF))*20))
test1.imp$Hb <- impute(test1.imp$Hb,
                        sum(test1_preimp_imp$imp$Hb)/(sum(is.na(test1_preimp$Hb))*20))
test1.imp$PLT <- impute(test1.imp$PLT,
                         sum(test1_preimp_imp$imp$PLT)/(sum(is.na(test1_preimp$PLT))*20))
test1.imp$WBC <- impute(test1.imp$WBC,
                          sum(test1_preimp_imp$imp$WBC)/(sum(is.na(test1_preimp$WBC))*20))
test1.imp$NA. <- impute(test1.imp$NA.,
                         sum(test1_preimp_imp$imp$NA.)/(sum(is.na(test1_preimp$NA.))*20))
test1.imp$k <- impute(test1.imp$k,
                      sum(test1_preimp_imp$imp$k)/(sum(is.na(test1_preimp$k))*20))
test1.imp$MG <- impute(test1.imp$MG,
                      sum(test1_preimp_imp$imp$MG)/(sum(is.na(test1_preimp$MG))*20))
test1.imp$ALT <- impute(test1.imp$ALT,
                     sum(test1_preimp_imp$imp$ALT)/(sum(is.na(test1_preimp$ALT))*20))
test1.imp$UA <- impute(test1.imp$UA,
                     sum(test1_preimp_imp$imp$UA)/(sum(is.na(test1_preimp$UA))*20))
test1.imp$ALB <- impute(test1.imp$ALB,
                     sum(test1_preimp_imp$imp$ALB)/(sum(is.na(test1_preimp$ALB))*20))
```

```
test1.imp$INR <- impute(test1.imp$INR,
                    sum(test1_preimp_imp$imp$INR)/(sum(is.na(test1_preimp$INR))*20))
test1.imp$LDL <- impute(test1.imp$LDL,
                    sum(test1_preimp_imp$imp$LDL)/(sum(is.na(test1_preimp$LDL))*20))
test1.imp$TIDL <- impute(test1.imp$TIDL,
                    sum(test1_preimp_imp$imp$TIDL)/(sum(is.na(test1_preimp$TIDL))*20))

write.csv(test1.imp, file ="test1.imp.csv") testdata1 <- read.csv("test1.imp.csv") ##---some
continuous variables analyzedas categorical variables---##

##---ROC in testing data---##
library(ROCR)
probs.test <- predict(fit1, newdata = testdata1, type = "response")
pred.full.test <- prediction(probs.test, testdata1$AKI)
perf.full.test <- performance(pred.full.test, "tpr", "fpr")
plot(perf.full.test, main = "ROC", col = "red")
performance(pred.full.test, "auc")@y.values

##----calibration curve in testing data----##
library(rms) val.prob(probs.test,
testdata1$AKI)

###---decision curve analysi in testing data----## install.packages("rmda") library(rmda)
simple<decision_curve(AKI~age+male+EF+Hypertension.histroy+preACEI+Hb+UA+MG+preNSAID
, data = testdata1, family = binomial(link ='logit'), thresholds= seq(0,1, by = 0.01), study.design =
'case-control', population.prevalence = 0.244, confidence.intervals = 0.95)

plot_decision_curve(list(simple),curve.names= c('nomangram'),
                              cost.benefit.axis =F,col = c('red'),
                              confidence.intervals =F,standardize = F)
```
