# Supplementary material for: Derivation and validation of a model to predict acute kidney injury following cardiac surgery in patients with normal renal function
Source: Ren Fail. 2021 Aug 9;43(1):1205–13. doi: 10.1080/0886022X.2021.1960563 (PMC8354173; doi:10.1080/0886022X.2021.1960563)
Supplement: Supplemental Material [file IRNF_A_1960563_SM7612.pdf]

Table S1. Univariate analysis between candidate variables and AKI in training group.

Variables	no-AKI (n=11746)	AKI (n=3955)	*P* value
Age, years	45.0 ± 14.2	52.7 ± 12.7	<0.001
Male	5429 (46.2%)	2282 (57.7%)	<0.001
LVEF			<0.001
LVEF >60%	9282 (79.0%)	2144 (54.2%)	
40%< LVEF ≤60%	2305 (19.6%)	1546 (39.1%)	
LVEF ≤40%	159 (1.4%)	265 (6.7%)	
Baseline serum creatinine, μmol/L	74.9 ± 17.0	77.2 ± 17.9	<0.001
eGFR, ml/min/1.73m^2	95.6 ± 19.3	89.9 ± 18.6	<0.001
Comorbidities			
Hypertension	1962 (16.7%)	1222 (30.9%)	<0.001
Diabetes mellitus	482 (4.1%)	309 (7.8%)	<0.001
Coronary heart disease	885 (7.5%)	619 (15.7%)	<0.001
COPD	149 (1.3%)	78 (2.0%)	0.001
Infectious endocarditis	634 (5.4%)	214 (5.4%)	0.970
Cerebrovascular disease	463 (3.9%)	223 (5.6%)	<0.001
Peripheral vascular disease	41 (0.3%)	24 (0.6%)	0.029
Atrial fibrillation	2883 (24.5%)	1190 (30.1%)	<0.001
PCI history	100 (0.9%)	59 (1.5%)	<0.001
Previous cardiac surgery	311 (2.6%)	143 (3.6%)	0.002
History of transfusion	24 (0.2%)	11 (0.3%)	0.390
Recent contrast media exposure	2443 (20.8%)	1100 (27.8%)	<0.001
Preoperative drugs use			
Renin-angiotensin system inhibitors	3437 (29.3%)	1824 (46.1%)	<0.001
NSAID	1434 (12.2%)	768 (19.4%)	<0.001
Aminoglycoside antibiotics	666 (5.7%)	245 (6.2%)	0.220
Stain	1226 (10.4%)	719 (18.2%)	<0.001
Proton pump inhibitors	4795 (40.8%)	1663 (42.0%)	0.180
Erythrocyte transfusion, U	0.0 (0.0, 0.0)	0.0 (0.0, 0.0)	0.002
Procedure			<0.001
CABG	460 (3.9%)	269 (6.8%)	
Valve	7217 (61.4%)	2573 (65.1%)	
Aortic	661 (5.6%)	450 (11.4%)	
CHD	2998 (25.5%)	393 (9.9%)	
CABG + valve	217 (1.8%)	239 (6.0%)	
Others	193 (1.6%)	31 (0.8%)	
Laboratory Findings			
Hemoglobin, g/L	135.7 ± 17.6	129.6 ± 18.2	<0.001
Platelet <100×10^9/L	291 (2.5%)	140 (3.5%)	<0.001
Blood leucocytes (×10^9/L)	7.1 ± 2.6	7.3 ± 2.6	<0.001
Natremia <135 mmol/L	664 (5.7%)	321 (8.1%)	<0.001

Potassium < 3.5mmol/L	1781 (15.2%)	737 (18.6%)	<0.001
Hypomagnesemia	2381 (20.3%)	1014 (25.6%)	<0.001
Alanine aminotransferase > 40 U/L	1295 (11.0%)	531 (13.4%)	<0.001
Uric acid, μmol/L	383.6 ± 102.6	409.5 ± 110.7	<0.001
Albumin, g/L	38.1 ± 3.8	37.2 ± 3.9	<0.001
International Normalized Ratio >1.5	1132 (9.6%)	481 (12.2%)	<0.001
Low density lipoprotein, mmol/L	3.0 ± 0.8	2.9 ± 0.8	<0.001
Total bilirubin >17.3μmol/L	5674 (48.3%)	1935 (48.9%)	0.500

CABG: coronary artery bypass grafting; CHD: congenital heart disease; COPD: chronic obstructive pulmonary disease; eGFR: estimated glomerular filtration rate; LVEF: left ventricular ejection fraction; NSAID: non-steroidal anti-inflammatory drugs; PCI: percutaneous coronary intervention.
